# Paleodistribution of Cercidiphyllaceae and Future Habitat Prediction for *Cercidiphyllum japonicum* Under Climate Change

**DOI:** 10.1002/ece3.72940

**Published:** 2026-01-19

**Authors:** Ping Mao, Min Zeng, Jiaxing Lv, Juan Wei, Qiuxia Feng, Yumin Shu, Yonghong Ma

**Affiliations:** ^1^ College of Life Science China West Normal University Nanchong China; ^2^ Sichuan Zhongtou Ecological Environment Technology Co. Ltd Chengdu China; ^3^ Chongqing Wansheng Economic and Technological Development Zone Teachers' Further Education School Chongqing China; ^4^ Key Laboratory of Southwest China Wildlife Resources Conservation (Ministry of Education) China West Normal University Nanchong China

**Keywords:** Cercidiphyllaceae, *Cercidiphyllum japonicum*, climate change, fossil, MaxEnt, potentially suitable habitats

## Abstract

The Earth's environment is an important factor driving the evolution and distribution of biodiversity, with particular regard to endangered species, whose special evolutionary history and ecological environment changes profoundly impact their distribution and even survival. This paper conducts a preliminary analysis of the coupling relationship between the geological history distribution pattern of plants in the Cercidiphyllaceae, a unique East Asian group, and paleoclimatic changes, exploring the evolution of Cercidiphyllaceae's geographic distribution pattern. The MaxEnt model was used to construct the potentially suitable habitats for *Cercidiphyllum japonicum* in different periods, such as the current and future (2050s and 2070s). Research shows that Cercidiphyllaceae once exhibited relatively high diversity, with 21 fossil species assigned to 5 fossil genera. From the Late Cretaceous to the Eocene, when the global paleotemperature was relatively high, they were widely distributed in the mid‐high latitude regions of the Northern Hemisphere. Since the Oligocene, with the global temperature decline, the number of species of Cercidiphyllaceae has decreased sharply, and the distribution habitats have also migrated to lower latitudes. Especially after experiencing multiple glacial periods in the Quaternary period, most species became extinct. Currently, only two species of the genus *Cercidiphyllum* remain, namely, *C. japonicum* and *Cercidiphyllum magnificum*, which are only discontinuously distributed in China and Japan. Under the current climatic conditions, the suitable habitat area of *C. japonicum* in China is 1,316,200 km^2^, primarily concentrated in the Hengduan Mountains and Qinling‐Daba Mountains. However, as temperatures rise because of global warming, the plant's viable habitat is projected to shrink significantly. In the 2050s and 2070s, the lightest contraction and the largest suitable habitat area are under the RCP6.0 climate scenario; in contrast, the most severe contraction and the smallest suitable habitat area are under the RCP4.5 climate scenario. These findings offer valuable insights for conservation efforts targeting this species, as well as other endangered plant species facing similar threats.

## Graphical abstract

Employing MaxEnt modeling, this research projects shifts in the potential suitable habitat range of *Cercidiphyllum japonicum* under climate change trajectories. Analysis identifies temperature and precipitation as dominant drivers governing habitat distribution, with models indicating significant range contraction throughout the forecast period.

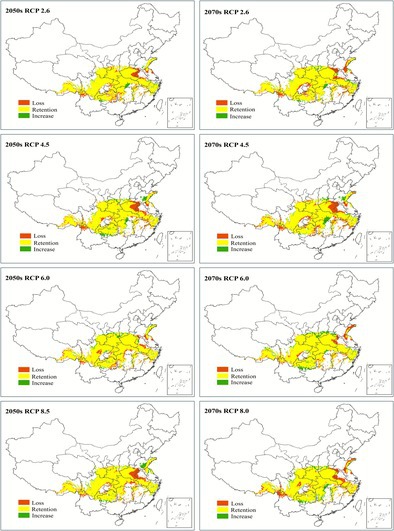

## 1 Introduction

Climate change is recognized as a key driver of species' range dynamics (Bai et al. 2018; Pearson and Dawson 2003; Puchałka et al. 2023). The glacial episodes of the Quaternary period (2.6 million years ago to present) were a major factor in shaping present‐day species distributions, whereas they led to the extinction of some terrestrial species, others survived by evolving adaptively in situ or by retreating to refugia (Provan and Bennett 2008). Climate shifts have caused considerable alterations in the spatial ranges of numerous species (Zhang et al. 2018; Zhao et al. 2021). The distribution range of plant species is primarily influenced by climatic factors, which also shift geographically because of climate change (Dieleman et al. 2015; Ruiz‐Labourdette et al. 2012). Therefore, combining the historical distribution of species and predicting the future distributions of species will help us better understand the response of species to climate change and the historical causes of species differentiation and formation, thus devising effective conservation strategies (Fan et al. 2022; Bai et al. 2018).

Species distribution models (SDMs) are one of the most important tools in ecological research, and such models are also the main tools used to predict the impact of climate change on species distribution (Booth 2018). Among numerous models, SDMs include 19 different methods, such as the rule‐set genetic algorithm model (GARP), maximum entropy model (MaxEnt), and ecological factor analysis model (ENFA) (Phillips and Dudik 2008). Research indicates that the maximum entropy model (MaxEnt) outperforms other models in terms of the accuracy of prediction results and has achieved good simulation effects, particularly when dealing with incomplete data on species distributions (Yi et al. 2017; Saatchi et al. 2008; Phillips et al. 2006). Elith et al. (2006) used 16 SDMs to predict the potential distributions of 226 species and found that MaxEnt significantly outperformed other models in accuracy.

*Cercidiphyllum japonicum* Sieb. et Zucc., is a Tertiary relict plant with a disjunct distribution between China and Japan (Qi et al. 2012). It holds significant scientific value for elucidating the origin of the Tertiary flora and the relationship between the Chinese and Japanese floristic regions. Unfortunately, its natural regeneration is poor. This is due to factors including low seed production, small and lightweight seeds, low stress tolerance, and difficult seed germination, coupled with a primary reliance on vegetative reproduction in wild populations, which leads to exceedingly slow population growth (Liu 2008). Therefore, it has been listed as a National Second‐level Key Protected Wild Plant in China (https://www.gov.cn/zhengce/zhengceku/2021‐09/09/content_5636409.htm).

*C. japonicum* was once distributed throughout the Northern Hemisphere, and fossil evidence indicates that *Cercidiphyllum* served as a major component of the forests in the Northern Hemisphere during the Late Cretaceous and the Tertiary (Crane and Stockey 1985). Lv (2021) demonstrated that the phenotypic variation in *C. japonicum* is closely correlated with corresponding environmental factors, such as longitude, altitude, temperature, and annual sunshine hours. Separately, Zeng et al. (2020) found that climatic factors significantly influence its distribution and noted that the population characteristics of *C. japonicum* exhibit a strong correlation with these climatic factors during the early growth stages. However, the specific influence of these climatic factors on the geographic distribution of *C. japonicum* remains ambiguous. What is the relationship between its current distribution pattern and historical climate change? How will its distribution pattern evolve in the context of future climate changes? What are the primary climatic factors constraining its geographical distribution, and how do these factors influence its distribution? These questions remain unanswered, impeding the effective protection and management of *C. japonicum* germplasm resources.

In this study, we compiled the geological record of the Cercidiphyllaceae, which comprises multiple genera in the fossil record. We compared fossil species across periods to examine the responses of these different genera to climate change. Furthermore, the future distribution of suitable habitat for *C. japonicum* under climate change was projected using the MaxEnt model. The objectives of this study are to (1) analyze historical range shifts of Cercidiphyllaceae in the context of paleoclimatic changes, (2) identify the key climatic drivers of the current distribution of *C. japonicum* , and (3) project future range shifts of *C. japonicum* under multiple climate change scenarios.

## 2 Materials and Methods

### 2.1 Data Sources of Geological History Distribution

This paper's fossil data mainly comes from the “Fossil Angiosperms in China” and the Paleobiology Database (https://paleobiodb.org/; Cui et al. 2019). The collected information includes fossil species, geological epochs, and distribution locations. Relevant paleogeological and paleoclimatic data were sourced from literature databases such as CNKI (https://www.cnki.net/), Baidu Scholar (https://xueshu.baidu.com/), and Wanfang Data Knowledge Service Platform (http://www.wanfangdata.com.cn/index.html).

### 2.2 Data Collection, Processing of the Current Distribution Point Data, and Preparation of the Distribution Map

The distribution data of *C. japonicum* mainly come from the National Plant Specimen Resource Center (CVH, https://www.cvh.ac.cn/) of China and more than 300 relevant literatures (from literature databases such as CNKI, Baidu Scholar, and Wanfang Data). Approximately 1000 distribution records of *C. japonicum* have been collected. The collected content includes specific distribution locations and their latitude and longitude information, whereas those without latitude and longitude information were determined by satellite map positioning to determine the approximate latitude and longitude information (accurate to the county level). After excluding artificially introduced cultivation data and duplicate entries, a total of 464 valid records were obtained, with 444 distributed in China and 20 in Japan.

We downloaded the 1:1,000,000 scale map of China, the provincial administrative division maps of China, and the county‐level administrative division maps of each province in China from the National Geomatics Center of China (https://www.ngcc.cn/dlxxzy/gjjcdlxxsjk/). Additionally, we downloaded the map of Japan and its administrative division maps from the Database of Global Administrative Areas (https://gadm.org). Using administrative division maps of China and Japan as base maps, the distribution point data of *C. japonicum* were superimposed with ArcGIS 10.2 software. By defining a consistent geographic coordinate system, a global distribution map of *C. japonicum* was generated.

### 2.3 Acquisition and Processing of Climatic Data

19 climatic variables with a spatial resolution of 30″ were downloaded from the global climate database WorldClim (https://www.worldclim.org). This study focuses on climatic data for two future periods, namely, the 2050s (2041–2060) and the 2070s (2061–2080). The future climate data are those under four different “Representative Concentration Pathways (RCPs)” scenarios. Specifically, these scenarios are RCP 2.6, RCP 4.5, RCP 6.0, and RCP 8.5 (Van Vuuren et al. 2011; Xin et al. 2013).

Because of the correlation between various climate variables, it is necessary to screen the climate factors to better study the climate factors affecting the distribution of *C. japonicum* . Using R software, this study employed the MaxEnt model combined with jackknife testing and Variance Inflation Factor (VIF) analysis to evaluate 19 climate variables of the current period (1970–2000). Seven climatic factors were ultimately identified as the most influential determinants of *C. japonicum* distribution (Table 1).

**TABLE 1:** Climatic variables for habitat limiting factor analysis of *C. japonicum* .

Number	Bioclimatic variables	Description
1	Bio2	Mean diurnal temperature range
2	Bio4	Temperature seasonality
3	Bio6	Minimum temperature of the coldest month
4	Bio9	Mean temperature of the driest quarter
5	Bio12	Annual precipitation
6	Bio15	Precipitation seasonality
7	Bio19	Precipitation of the coldest quarter

### 2.4 Processing of Distribution Data

To avoid spatial autocorrelation of species distribution points, 444 collected distribution points were separated by a 5 km × 5 km grid. If there were multiple distribution points in one grid, it would indicate that the distribution points in this grid had spatial autocorrelation. By substituting the particle of each grid for the distribution point of the *C. japonicum* in the grid, 326 distribution points are obtained by substituting the center point of the grid. The information on the distribution points of *C. japonicum* after the re‐screening was saved in CSV format, which was used for the simulation and prediction of the potentially suitable habitats of *C. japonicum* by the MaxEnt model.

### 2.5 Model Building and Evaluation

Import the distribution points of *C. japonicum* in CSV format and the climatic data files in ASCII format into the MaxEnt model. Check the seven climatic variables, namely Bio2, Bio4, Bio6, Bio12, Bio15, and Bio19, which have been screened out, and use the bootstrap method to cross‐validate the model. Eighty percent of occurrence records were allocated for model training, with the remaining 20% reserved for model performance evaluation. Using jackknife testing, we quantified the contribution of each climatic variable to *C. japonicum* 's distribution, repeating the entire procedure 10 times (Shi et al. 2021). Model performance was assessed via the area under the curve (AUC), where values (0–1) reflect predictive accuracy (higher values indicating greater accuracy) (Etherington 2011). Following Gan et al. (2024), we classified AUC performance into five tiers: unqualified (0.5–0.6), poor (0.6–0.7), average (0.7–0.8), good (0.8–0.9), and excellent (0.9–1.0). Values closer to 1.0 denote optimal model performance.

### 2.6 Classification of Suitable Habitats，Area Statistics，and Area Change

The results of the potentially suitable habitats for *C. japonicum* output by the MaxEnt model are ASCII data. After converting it into raster data, each grid will have a value representing the suitable habitat index of *C. japonicum* in that grid, ranging from 0 to 1. From the item “Maximum training sensitivity plus specificity” in the output results of the MaxEnt model, it can be known that the threshold value of the potentially suitable habitats for *C. japonicum* is 0.171. The potentially suitable habitats for *C. japonicum* are divided according to this threshold value. The areas where the raster value is less than 0.171 are the unsuitable areas for *C. japonicum* , and the areas where the value is greater than or equal to 0.171 are suitable habitats for *C. japonicum* (Manel et al. 2001).

On the basis of the data of 326 effective distribution points and 7 future (2050s, 2070s) climatic factors, the MaxEnt model was used to predict the potentially suitable habitats for *C. japonicum* . Maps were created with ArcGIS 10.2 to compare potentially suitable habitats for *C. japonicum* under the current climatic scenario and future climatic scenarios. We determined the expansion, stability, and contraction ranges of *C. japonicum* 's potentially suitable habitats under different concentration pathways. Using ArcToolbox, we calculated the total area of these potentially suitable habitats, as well as the areas of expansion, stability, and contraction under each pathway. For assessing *C. japonicum* habitat shifts across climate scenarios, distribution changes were categorized as: retention (3), increase (2), and loss (1).

The dispersal ability of species can be divided into two extreme forms: unlimited and no dispersal (Araújo and Luoto 2007). The net change in potentially suitable habitat area under unlimited dispersal conditions was calculated as (Expansion Area—Contraction Area)/Current Suitable Habitat Area across different future concentration pathways. The change under no‐dispersal conditions was calculated as Contraction Area/Current Suitable Habitat Area.

## 3 Results

### 3.1 Evolutionary History and Paleodistribution of Cercidiphyllaceae

Cercidiphyllaceae represents an ancient plant group with an extensive geological history and abundant fossil records. A total of 21 fossil species belonging to 5 genera have been collected, primarily distributed in western North America and northeastern Asia (Table 2). As indicated in Table 2, *Trochodendroides* and *Nyssidium* represent the same plant (Zhou and Momohara 2005).

**TABLE 2:** Fossil records of *C. japonicum* .

Time interval	Fossil species	Ma	Country or state
Albian	Trochodendroides potomacensis	113.0–100.5	Canada (British Columbia, Alberta)
Albian‐Cenomanian	Trochodendroides arctica	113.0–93.9	Russian Federation
Late Cretaceous	Trochodendroides arctica	99.6–93.5	Canada (British Columbia, Alberta)
		89.8–72.1	Canada (British Columbia)
		85.8–83.5	Canada (Alberta)
		72.1–66.0	Canada (Alberta)
	Trochodendroides flabella	70.6–66.0	Canada (Saskatchewan)
	Cercidiphyllum genetrix	70.6–66.0	USA (North Dakota)
	Trochodendroides nebrascensis	70.6–66.0	USA (North Dakota)
	Cercidiphyllum ellipticum	100.5–66.0	USA (Wyoming)
		72.1–66.0	USA (North Dakota)
		70.6–66.0	USA (North Dakota, Wyoming)
	Cercidiphyllites brevicolpatus	83.5–70.6	Canada (Alberta)
	Cercidiphyllum arcticum		China (Heilongjiang)
	Cercidiphyllum crenatum		China (Jilin)
Paleogene	Trochodendroides flabella	66.0–61.7	USA (Montana)
		66.0–56.0	Canada (Saskatchewan)
		58.7–55.8	USA (Wyoming)
	Cercidiphyllum genetrix	66.0–63.3	USA (Wyoming, North Dakota)
		66.0–61.7	USA (Montana)
		63.8–60.9	USA (Wyoming, North Dakota)
		61.7–56.8	USA (Wyoming)
		58.7–55.8	USA (Wyoming)
	Cercidiphyllum genesevianum	66.0–56.0	Canada (Alberta)
		66.0–56.0	China (Heilongjiang)
	Cercidiphyllum diversifolium	66.0–56.0	China (Xinjiang)
			China (Jiayin)
	Cercidiphyllum flexuosum	66.0–56.0	Canada (Alberta)
	Archeampelos acerifolia	66.0–56.0	Canada (Saskatchewan)
	Nyssidium jiayinense		China (Jiayin)
Paleogene—Eocene	Cercidiphyllum genetrix	56.2–54.9	USA (North Dakota, Wyoming)
	Trochodendroides serrulata	56.2–54.9	USA (North Dakota)
Eocene	Trochodendroides arctica	55.8–48.6	Canada (British Columbia)
		47.8–37.71	China (Jilin)
	Cercidiphyllum genetrix	55.8–50.3	USA (Wyoming, North Dakota)
	Cercidiphyllum elongatum	56.0–33.9	China (Henan)
		55.8–48.6	USA (Idaho)
	Cercidiphyllum obtritum	56.0–47.8	Canada (British Columbia)
		47.8–38.0	USA (Washington)
	Cercidiphyllum apocynoides	56.0–47.8	United Kingdom (England)
	Cercidiphyllum arcticum		China (Liaoning, Jilin)
Oligocene	Cercidiphyllum crenatum	33.9–28.4	USA (Oregon)
		33.9–28.1	USA (Idaho), Czech Republic
		28.4–15.97	USA (Montana)
		28.1–20.44	Germany (Sachsen)
	Cercidiphylloxylon kadanense		Czech Republic
Miocene	Cercidiphyllum crenatum	23.03–5.333	China (Inner Mongolia)
		23.03–2.588	Japan (Nagano)
		15.97–13.65	Switzerland (Zürich)
		15.97–5.333	Japan (Hirakata)
		13.82–11.62	Germany (Brandenburg)
	Cercidiphyllum elongatum	23.03–23.03	USA (Montana)
	Cercidiphyllum helveticum	15.97–13.82	Germany (Brandenburg)
		13.82–11.62	Germany (Brandenburg)
Pliocene	Cercidiphyllum crenatum	3.6–2.588	Germany (Thuringia)
Paleogene‐Neogene	Cercidiphyllum crenatum	66.0–2.58	Nagano, Japan
Pleistocene	*Cercidiphyllum japonicum*	2.588–0.781	Japan (Tokyo)

*Trochodendroides potomacensis* is the oldest fossil record discovered in Alberta and British Columbia, Canada, dating back approximately 113 Ma. However, only two additional fossil species, both of *Trochodendroides*, were found over the subsequent 10 million years. Fossil records from the late Cretaceous include 8 species belonging to 3 genera (*Trochodendroides*, *Cercidiphyllum*, and *Cercidophyllites*), which are primarily found in the western regions of the United States and Canada, as well as in Northeast China. In the Paleocene (66.0–56.0 Ma), seven fossil species belonging to 3 genera were discovered, and one new genus (*Archeampelos*) was established. Although there was no significant change in the main distribution areas, the fossil species *Cercidiphyllum diversifolium* was first found in Xinjiang, China, during this period. Six Eocene fossil species, belonging to *Cercidiphyllum* and *Trochodendroides*, were primarily distributed across northeastern China, western Canada, and the northwestern United States. Additionally, the fossil species *Cercidiphyllum apocynoides* was discovered in southern England.

Since the Oligocene, the number of Cercidiphyllaceae fossil species has sharply decreased, and the distribution center has gradually shifted from high latitudes to low latitudes. Only two new fossil species have been discovered over the 30 million years from the Oligocene to the Pleistocene. Fossils of these species also began to appear more widely in central Europe and eastern Asia, ultimately remaining only in East Asia. The extant genus *Cercidiphyllum* first appeared during the Maastrichtian stage of the Late Cretaceous (70.6–66.0 Ma). By the Pleistocene (2.588–0.781 Ma), 12 fossil species have been recorded. Currently, *C. japonicum* and *C. magnificum* are the only two species that survive in this genus and are only distributed in China and Japan.

### 3.2 Current Distribution Status of *C. japonicum*

**FIGURE 1:**
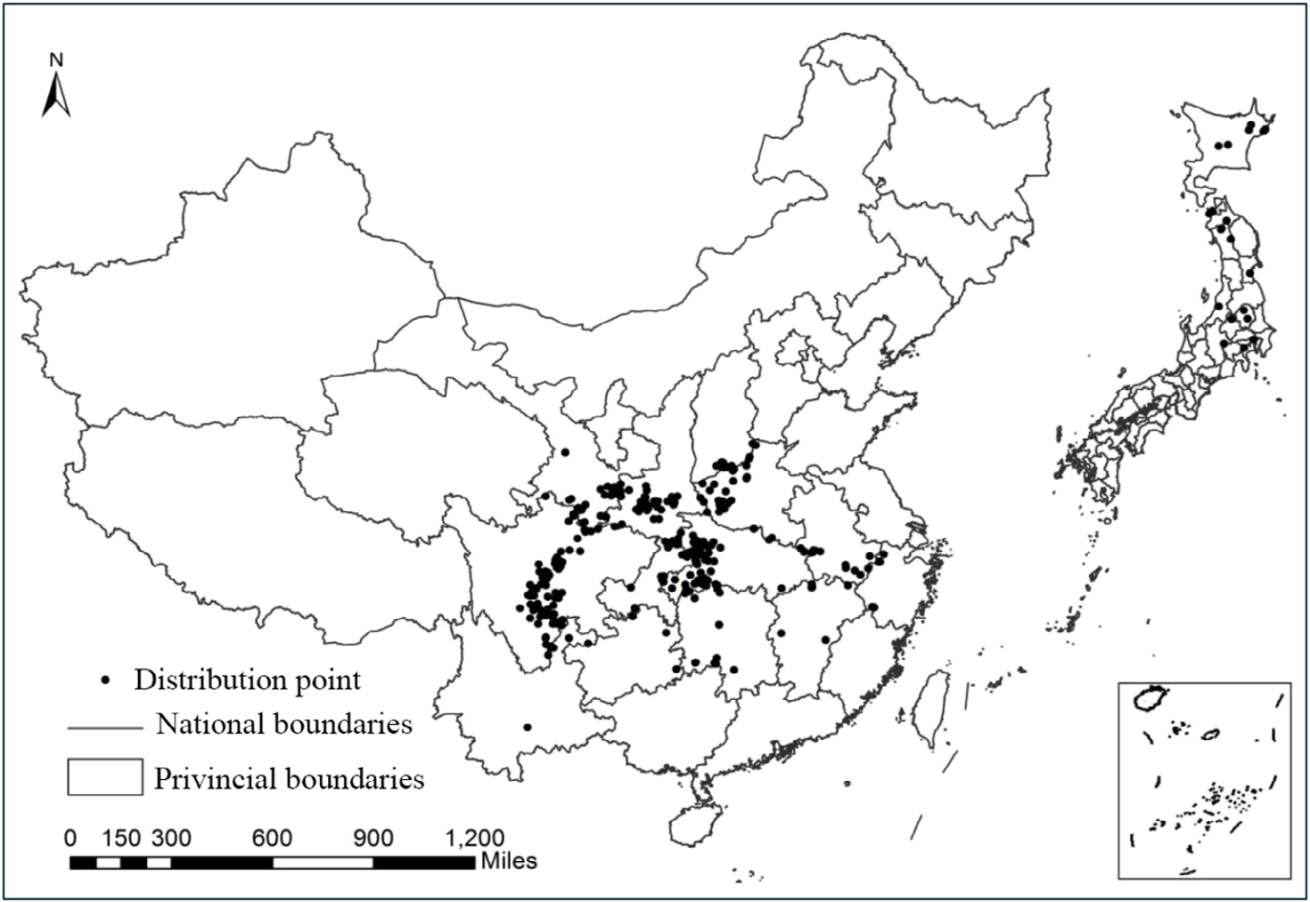
Global geographical distribution of *C. japonicum*.

In China, it is primarily distributed in the southeastern and southwestern regions. Its main distribution areas include central Sichuan, southern Gansu, southern Shaanxi, western Henan, and southwestern Hubei, with scattered occurrences in Chongqing, Yunnan, Guizhou, Hunan, Jiangxi, Anhui, and Zhejiang. In Japan, *C. japonicum* is primarily found in the Hokkaido, Tohoku, Kanto, and Chubu regions (Figure 1).

### 3.3 Accuracy of the Maxent Model

**FIGURE 2:**
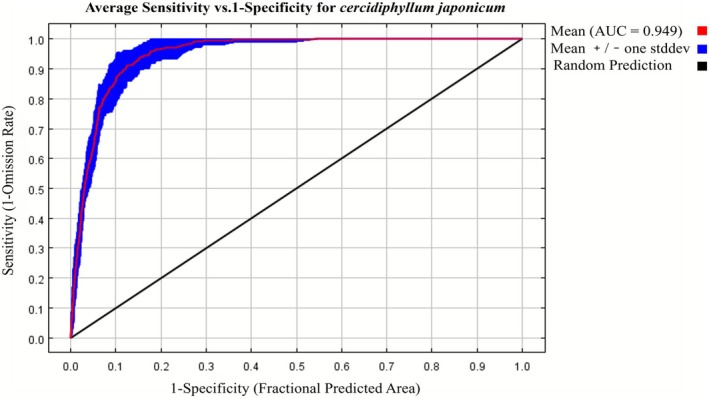
ROC curve.

The area under the ROC curve (AUC value) was used to evaluate the model's accuracy. After 10 iterations, the average AUC value of the current suitable habitat model was 0.949 (Figure 2). This demonstrates that the MaxEnt model exhibits outstanding simulation performance in depicting the geographical distribution of the potentially suitable habitats for *C. japonicum* and possesses high credibility.

### 3.4 Analysis of the Main Climatic Factors

Jackknife testing of seven environmental factors revealed Bio12 as the top contributor (33.2%), followed by Bio6 at 29.6%. This analysis revealed that Bio12 and Bio6 mainly influence the present distribution of *C. japonicum* (Table 3).

**TABLE 3:** Relative contribution percentage of climate variables to the MaxEnt model.

Bioclimatic variables	Description	Percent contribution
Bio12	Annual precipitation	33.2
Bio6	Minimum temperature of the coldest month	29.6
Bio9	Mean temperature of the driest quarter	13.5
Bio4	Temperature seasonality	13
Bio2	Mean diurnal temperature range	7.1
Bio19	Precipitation of the coldest quarter	2.2
Bio15	Precipitation seasonality	1.4

**FIGURE 3:**
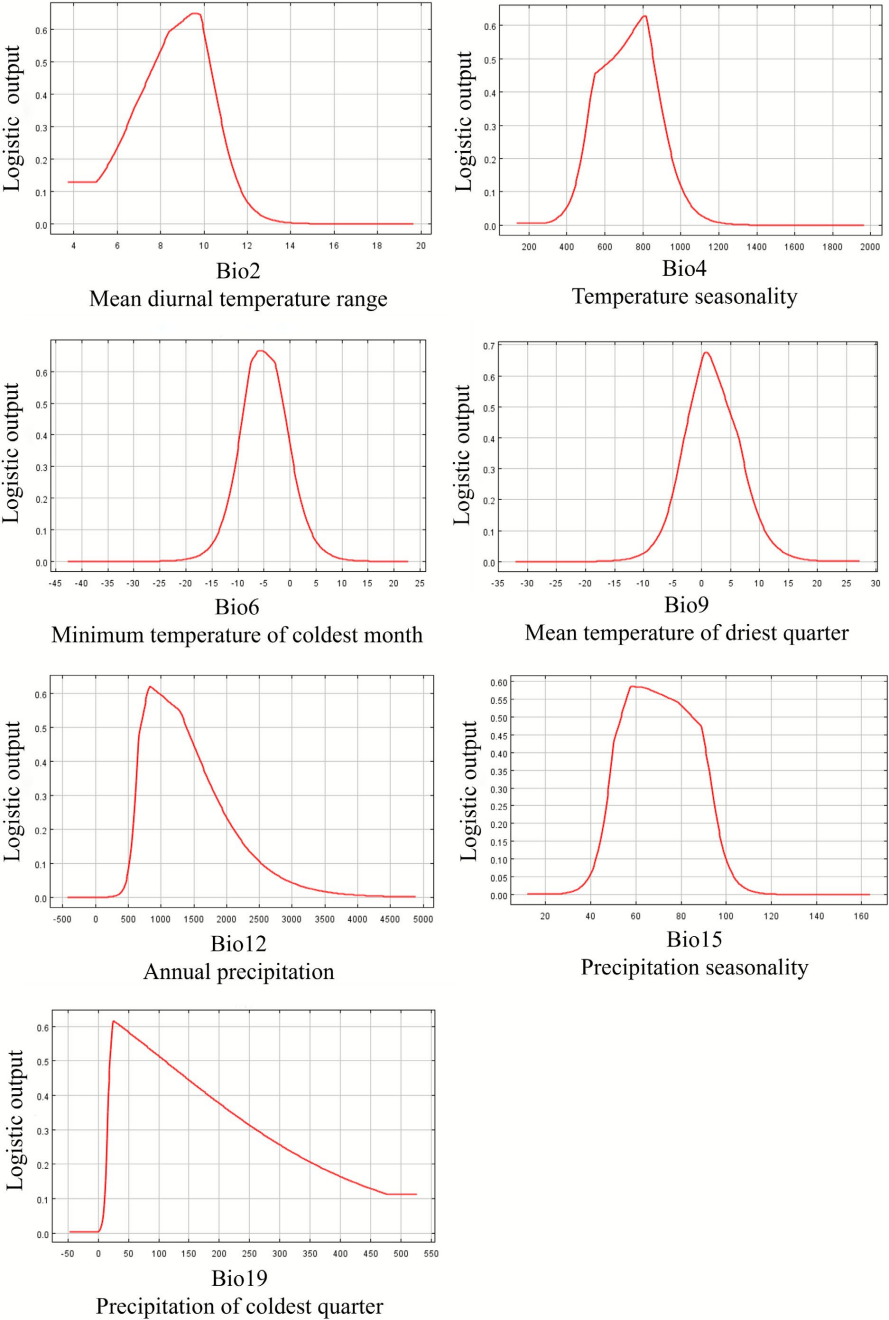
Response curves of various climatic factors.

Environmental factor response curves further elucidated the relationship between the probability of *C. japonicum* occurrence and environmental variables (Figure 3). The results show that all climatic factors exhibit a unimodal response pattern. When Bio2 is between 8°C and 10°C, Bio6 is between −8°C and −2°C, and Bio9 is between 0°C and 3°C, the occurrence probability of *C. japonicum* peaks. The optimal value of Bio4 is approximately 800. When Bio12 falls below 500 mm, the occurrence probability approaches zero, with an optimum at approximately 800 mm. The occurrence probability peaks when Bio15 ranged between 50 and 90, and is highest for Bio19 at approximately 25 mm, declining progressively beyond this value.

### 3.5 Suitable Distribution Habitats for *C. japonicum* Under Current Climatic Conditions

**FIGURE 4:**
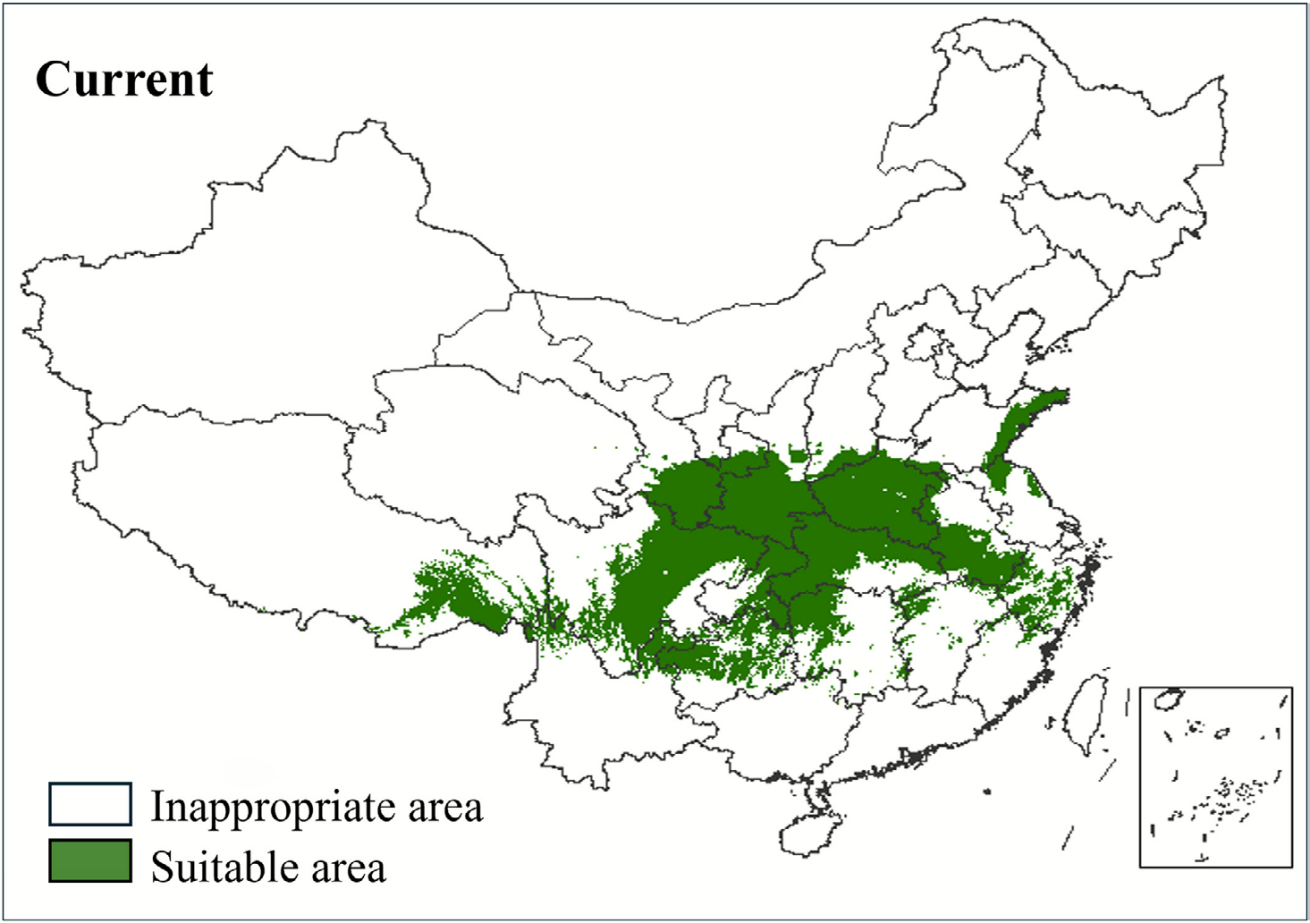
Potentially suitable habitats for *C. japonicum* in the current period.

Suitable habitats for *C. japonicum* are predominantly found across central Sichuan, northern Guizhou, southern Gansu, southern Shaanxi, eastern Chongqing, most of Hubei and Henan, and southern Anhui, with limited occurrence in neighboring regions. The primary suitable habitats are concentrated in three key regions: (1) the Hengduan Mountains, (2) the Qinling‐Daba Mountains, and (3) the mountainous regions of Hubei, Chongqing, and Hunan (Figure 4). The currently suitable habitat area for *C. japonicum* spans 1,316,200 km^2^, accounting for 13.71% of China's total land area (Table 4).

**TABLE 4:** Changes of suitable habitat area for *C. japonicum* in different periods (km^2^).

Period	Area	Increase	Stable	Decrease	Change
Current	1,316,200	—	—	—	—
2050s RCP2.6	1,221,125	84,250	1,136,875	179,325	−95,075
2050s RCP4.5	1,211,950	126,475	1,085,475	230,725	−104,250
2050s RCP6.0	1,261,025	98,025	1,163,000	153,200	−55,175
2050s RCP8.5	1,215,050	102,950	1,112,100	204,100	−101,150
2070s RCP2.6	1,209,250	93,875	1,115,375	200,825	−106,950
2070s RCP4.5	1,144,250	107,800	1,036,450	279,750	−171,950
2070s RCP6.0	1,307,075	157,225	1,149,850	166,350	−9125
2070s RCP8.5	1,215,675	125,925	1,089,750	226,450	−100,525

*Note:* Positive number means the area of suitable area increases; negative number means the area of suitable area decreases.

### 3.6 Prediction of Suitable Habitats for *C. japonicum* in Future Climates

**FIGURE 5:**
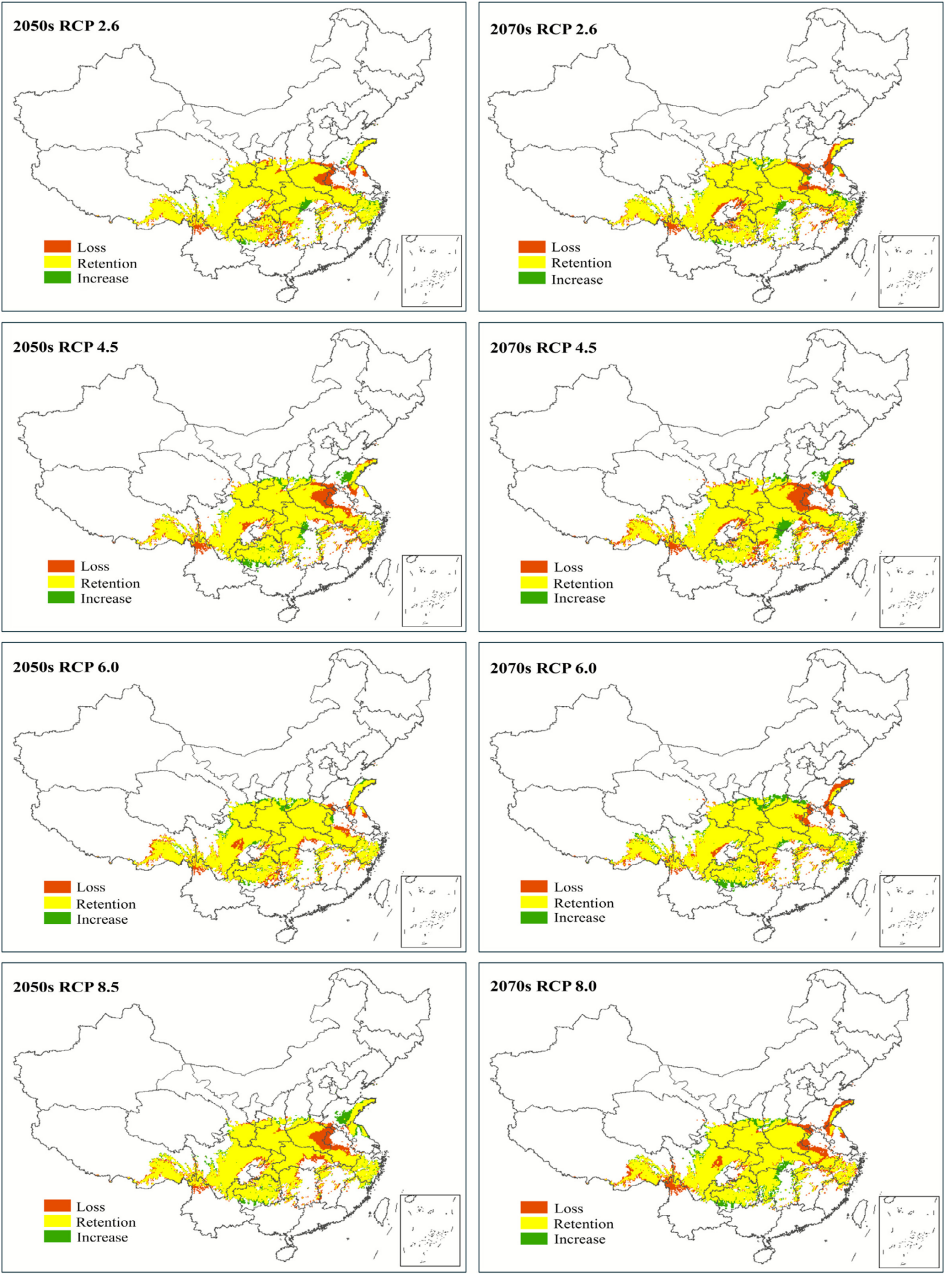
Changes in the range of potentially suitable habitats for *C. japonicum* in the future.

The range of suitable habitats for *C. japonicum* under different future concentration pathways remains unchanged compared with the current situation. Suitable habitats remain concentrated in Southwest and Central China, with additional distribution in Northwest and East China. Contractions primarily occur in the border regions of Anhui and Henan provinces, as well as northwestern Yunnan. At the same time, expansions are mainly observed in Guizhou, Shandong, and the Hubei‐Hunan border area. Notably, the contracted area of suitable habitats is significantly larger than the expanded area (Figure 5).

In the four 2050s scenarios, the potentially suitable habitat area under RCP 2.6, RCP 4.5, RCP 6.0, and RCP 8.5 is 1,221,125 km^2^, 1,211,950 km^2^, 1,261,025 km^2^, and 1,215,050 km^2^, respectively. Compared to current climatic conditions, these represent contractions of 95,075 km^2^, 104,250 km^2^, 55,175 km^2^, and 101,150 km^2^, respectively. Among these scenarios, RCP 6.0 maintains the largest habitat area with the least shrinkage, whereas RCP 4.5 shows the smallest habitat area with the most significant shrinkage. In the four 2070s scenarios, the potentially suitable habitat area under RCP 2.6, RCP 4.5, RCP 6.0, and RCP 8.5 is 1,209,250 km^2^, 1,144,250 km^2^, 1,307,075 km^2^, and 1,215,675 km^2^, respectively. Compared to current climatic conditions, these represent contractions of 106,950 km^2^, 171,950 km^2^, 9125 km^2^, and 100,525 km^2^. Among these scenarios, RCP 6.0 still maintains the largest habitat area with the least shrinkage, whereas RCP 4.5 also shows the smallest habitat area with the most significant shrinkage (Table 4).

**FIGURE 6:**
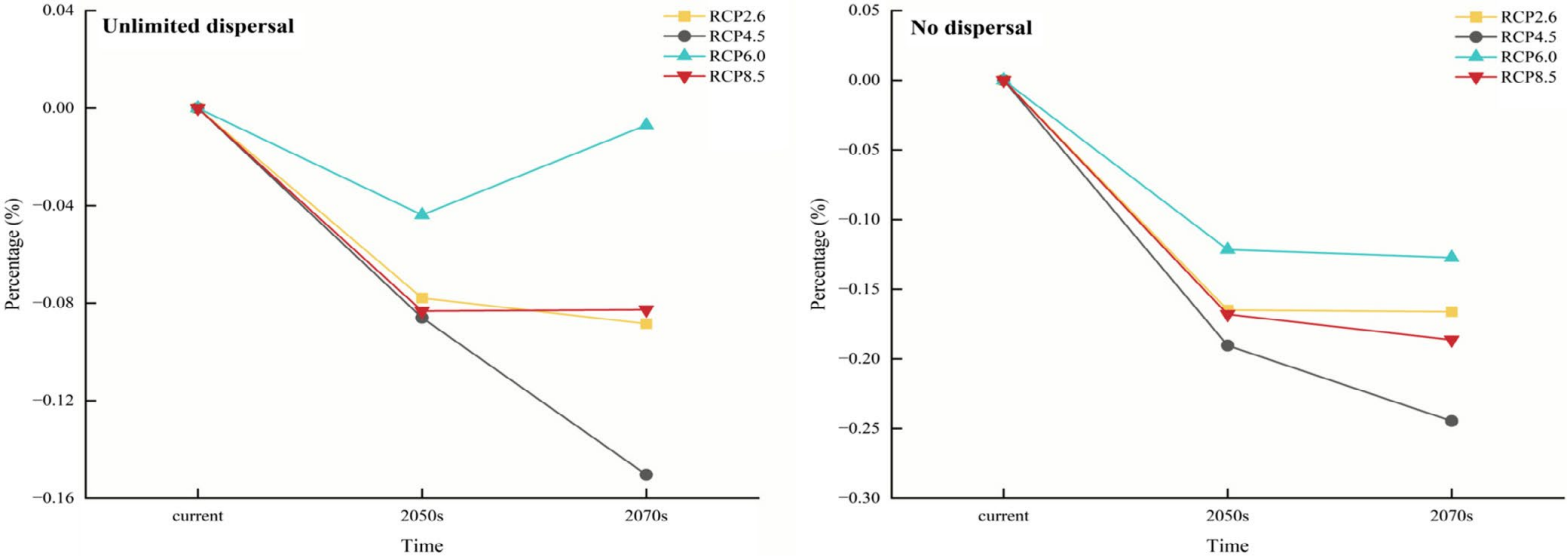
Area change of potentially suitable habitats for *C. japonicum* with time.

One of the key factors influencing species' distribution is the species' dispersal ability (Russell et al. 2015). The dispersal ability of species can be divided into two extreme forms: unlimited and no dispersal. Species with strong dispersal capabilities approximate the unlimited dispersal scenario, whereas those with weak dispersal approach the no‐dispersal condition. Under unlimited dispersal conditions, *C. japonicum* habitats exhibit both contraction and expansion. Conversely, under no‐dispersal conditions, only habitat contraction occurs without expansion (Figure 6).

## 4 Discussion

### 4.1 Environmental Factors Influencing the Geographical Distribution of *C. japonicum*

Jackknife testing revealed that Bio12 and Bio6 are the most important climatic variables in the MaxEnt model for predicting suitable *C. japonicum* habitat. Therefore, temperature and precipitation are the primary drivers of *C. japonicum* 's geographical distribution, a finding consistent with prior research. For example, Zeng et al. (2020), when simulating the potentially suitable habitats for *C. japonicum* in China, found that climatic factors and topographic factors jointly affect the distribution of *C. japonicum* . The annual average precipitation, the minimum temperature of the coldest month, the slope, and the altitude have the greatest impact on the potentially suitable habitats for *C. japonicum* . Similarly, employing the Maxent model, Hao and Zhou (2024) predicted *C. japonicum* 's potential distribution in Shanxi, identifying temperature and precipitation as the dominant influences. Threshold analysis indicates *C. japonicum* thrives better in environments with sufficient precipitation and low temperatures with less rainfall during winter, reinforcing conclusions from prior studies.

### 4.2 Shifts in the Geographical Distribution of *C. japonicum*

The results indicate that both the diversity and distribution of fossil Cercidiphyllaceae species exhibit a significant correlation with paleotemperature. Species diversity and lineage differentiation levels were substantially higher during warmer geological periods than during cooler global temperature regimes. Fossil records of Cercidiphyllaceae first appeared in the Early Cretaceous and were prevalent in mid‐ to high‐latitude regions of the Northern Hemisphere during the Tertiary. Shifting geographical and climatic conditions subsequently reduced the distribution range of Cercidiphyllaceae, with its core distribution area ultimately shifting from North America to the Eurasian continent. During the Late Cretaceous to Paleocene, the global climate was relatively warm, with mid‐ to high‐latitude regions characterized by warm, humid conditions (Yu 1995). Consequently, Cercidiphyllaceae plants—adapted to such environments—were distributed throughout these Northern Hemisphere latitudes. During the Early Tertiary, warm and humid conditions prevailed across the Northern Hemisphere, sustaining a broad distribution of flora in mid‐high latitudes, including Arctic regions (Tiffney 1985). By the Eocene, although global temperatures remained elevated, Cercidiphyllaceae plants exhibited limited range expansion—remaining primarily confined to North America and mid‐high latitude zones of Eurasia (Inglis et al. 2020). The Eocene–Oligocene Transition (EOT) is a global rapid cooling event. It is characterized by a rapid transition of the Earth's climate from the previous warm and humid period (Green House) to the Ice House period, with the emergence of an ice sheet in Antarctica (i.e., the Antarctic Ice Sheet), accompanied by a significant drop in global temperatures (Toumoulin et al. 2022; Sun et al. 2014). The cooling event at the Eocene/Oligocene boundary may be one of the reasons for the decrease in the fossils of Cercidiphyllaceae plants during the Oligocene period (Zanazzi et al. 2007). During the Middle‐Late Miocene, Earth's climate transitioned from a relatively warm phase to a colder mode with the reestablishment of permanent ice sheets on Antarctica. Because of the gradual decrease in temperature, the distribution range of Cercidiphyllaceae plants gradually shifted southward, similar to Zhang et al.'s (2025) research results (Holbourn et al. 2014; Steinthorsdottir et al. 2021). The Pliocene‐Pleistocene is a transition from the Tertiary to the Quaternary and a turning point in the climate from the “Greenhouse” to the “Icehouse”. Because of the sudden climate drop, Cercidiphyllaceae plants' diversity dropped sharply during these two periods.

Only two extant species of modern Cercidiphyllaceae plants are disjunctly distributed in China and Japan. The formation of the current China‐Japan distribution pattern of *C. japonicum* may be caused by the drastic climate changes in the Quaternary and the cold and harsh environment. The Quaternary period was characterized by distinct alternations between glacial and interglacial climates. The glacial phases were marked by a substantial decline in global temperatures, which facilitated the development of extensive ice sheets across mid‐to‐high latitudes and mountainous regions (Zhang 2022). The Quaternary glaciations exerted a particularly strong influence on Europe and North America. During the Last Glacial Maximum (LGM), vast land areas were buried under continental ice sheets, leading to the extinction of numerous plant species (Chen 2022). Compared with Europe and North America, East Asia only formed limited glacier coverage during the Quaternary glacial period (Fu and Wen 2023). During this period, suitable habitats for *C. japonicum* diminished, primarily contracting to southwest China. Mountain barriers attenuated cold air penetration, minimizing climate extremes and generating stable refugia that supported species survival (Li 2017; Stewart et al. 2010). The Quaternary glaciers only covered the northern part of Japan and the tops of mountainous areas, and glaciers did not invade most areas. Therefore, the Cercidiphyllaceae plants distributed in Japan were preserved, ultimately forming the current China‐Japan distribution pattern of *C. japonicum* (Liu et al. 2023).

In the future, global warming will significantly reduce suitable habitats for *C. japonicum* . Compared to current climatic conditions, the potentially suitable habitats under all scenarios show varying degrees of contraction. This results from temperatures surpassing the physiological optimum for *C. japonicum* growth. As a result, rising temperatures become the main factor limiting future suitable habitats. In addition, the reduction of suitable habitat area for *C. japonicum* observed under both unlimited dispersal and no‐dispersal conditions indicates that climate change will indeed negatively impact the distribution of this species.

## 5 Conclusions

Cercidiphyllaceae first appeared during the Early Cretaceous period. During the Tertiary period, they were extensively found across the northern hemisphere's middle and high latitudes. After the Quaternary glacial period, they were only distributed in some areas of China and Japan. From the Late Cretaceous to the Tertiary, excellent geographical and climatic conditions were provided for Cercidiphyllaceae plants' survival and evolution, and their distribution range gradually expanded. From the end of the Tertiary to the Quaternary, the climate deteriorated. The extensive coverage of glaciers led to the extinction of Cercidiphyllaceae plants in North America and Europe. The Cercidiphyllaceae plants in East Asia were only preserved in the “refuge” areas.

The main climatic variable influencing the distribution of suitable habitats for *C.japonicum* is Bio6 and Bio12. *C.japonicum* thrives better in environments with sufficient precipitation and low temperatures with less rainfall during winter.

Under current climate conditions, potentially suitable habitats for *C. japonicum* are concentrated in the Hengduan Mountains, Qinling‐Daba Mountains, and the mountainous regions of Hubei, Chongqing, and Hunan.

The range of suitable habitats for *C. japonicum* under future climatic conditions does not change much compared with the current situation. There are both expansions and contractions, but overall, it is in a state of contraction. Contractions primarily occur in the border regions of Anhui and Henan provinces, as well as northwestern Yunnan. Expansions are mainly observed in Guizhou, Shandong, and the Hubei‐Hunan border area. The area of the potentially suitable habitats for *C. japonicum* is shrinking both under the state of unlimited dispersal and no dispersal, indicating that climate change has negatively impacted its distribution.

## Author Contributions

**Ping Mao:** conceptualization (equal), data curation (equal), methodology (equal), software (equal), writing – original draft (equal). **Min Zeng:** data curation (equal), software (equal), visualization (equal). **Jiaxing Lv:** methodology (equal), software (equal). **Juan Wei:** methodology (equal), software (equal). **Qiuxia Feng:** methodology (equal), software (equal). **Yumin Shu:** data curation (equal), software (equal), visualization (equal). **Yonghong Ma:** data curation (equal), project administration (equal), supervision (equal), visualization (equal), writing – review and editing (equal).

## Funding

This work was supported by the Natural Science Foundation of Sichuan Province, 23NSFSC1272 Innovation Team Funds of China West Normal University, KCXTD2022‐4, CXTD2012‐7.

## Conflicts of Interest

The authors declare no conflicts of interest.

## Supporting information


**Data S1:** ece372940‐sup‐0001‐supinfo.zip.

## Data Availability

The required data are uploaded as Supporting Information.
